# Supplementary material for: Bottom-Up Proteomics Under Acidic Conditions Using Protease Type XIII From Aspergillus saitoi
Source: Mol Cell Proteomics. 2025 Nov 15;25(1):101469. doi: 10.1016/j.mcpro.2025.101469 (PMC12794574; doi:10.1016/j.mcpro.2025.101469)
Supplement: Supplemental data [file mmc1.pdf]

Supporting Information

Bottom-up proteomics under acidic conditions using protease type XIII from *Aspergillus saitoi*

Ryota Tomioka[1, 2], Ayana Tomioka[1], Kosuke Ogata[1], Yasushi Ishihama[1, 3*]

Contents:

Supplementary Figure S1 Total ion chromatograms after P13ase digestion of HeLa cell extract at various pH values
Supplementary Figure S2 Frequencies of missed cleavage by P13ase at various pH values
Supplementary Figure S3 Effect of digestion pH on artifactual deamidation and succinimidation
Supplementary Figure S4 Peptide lengths after P13ase digestion of HeLa cell extract for various times and at various temperatures
Supplementary Figure S5 Retention time distribution after P13ase digestion of HeLa cell extract for various times and at various temperatures
Supplementary Figure S6 Cleavage preferences of P13ase at various digestion times and temperatures
Supplementary Figure S7 Cleavage specificity of P13ase at various digestion temperatures and times
Supplementary Figure S8 Missed cleavage frequencies of P13ase at various digestion temperatures and times
Supplementary Figure S9 Effect of digestion temperature and time on artifactual deamidation and succinimidation
Supplementary Figure S10 Self-digested peptides generated by P13ase or trypsin
Supplementary Figure S11 Characteristics of peptides identified by P13ase digestion of HeLa cell extract
Supplementary Figure S12 Characteristics of proteins identified by P13ase digestion of HeLa cell extract
Supplementary Figure S13 Sequence coverage of histone H2A type 1 by P13ase or trypsin digestion

Supplementary Table S1 Lists of peptides and proteins identified in this study (as a separate .xlsx file)
Supplementary Table S2 Motif analysis of the cleavage sites of P13ase (pH 3.5)
Supplementary Table S3 Motif analysis of the cleavage sites of P13ase (37°C, 60 minutes)
Supplementary Table S4 Motif analysis of the cleavage sites of P13ase (37°C, 16 hours)

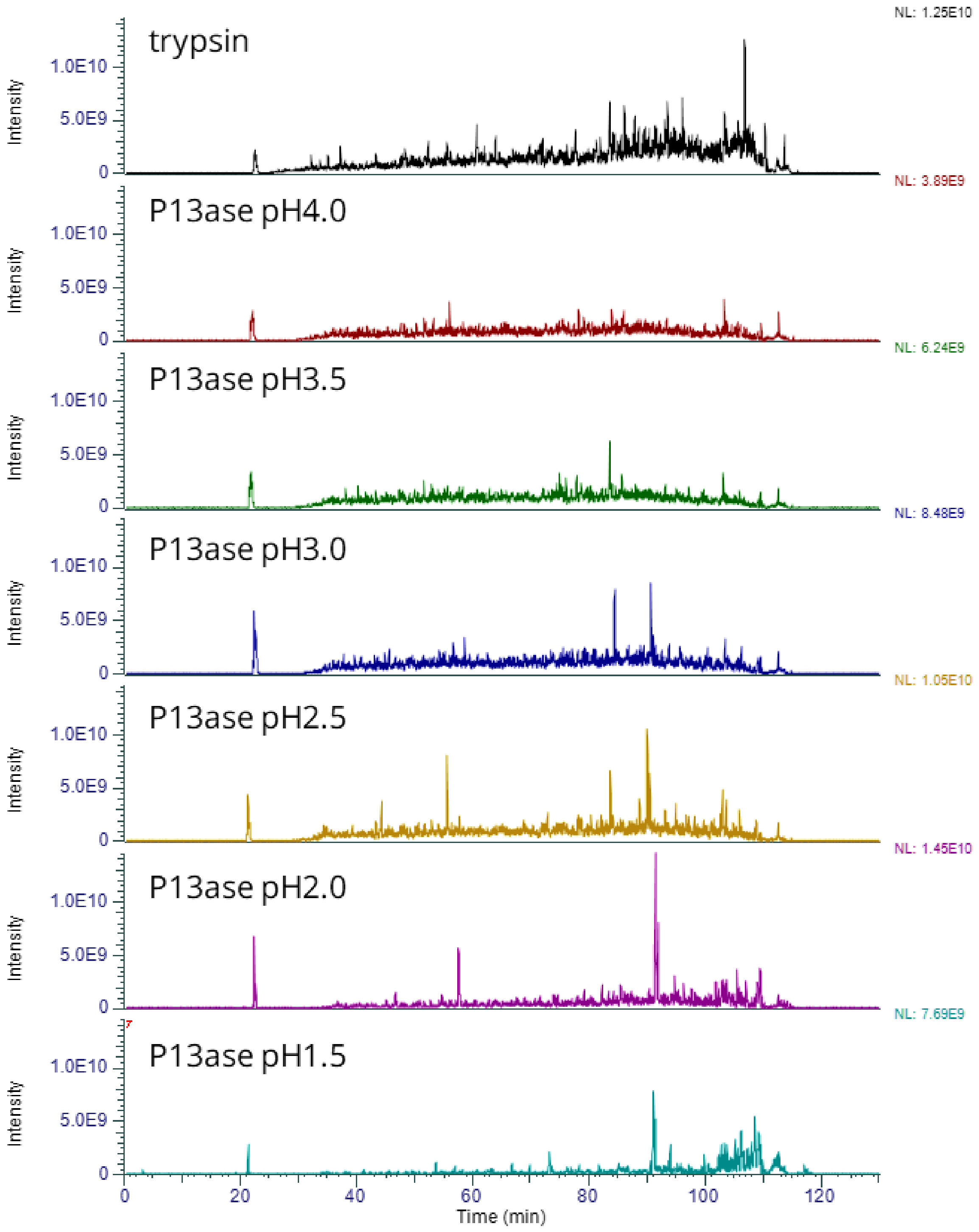

Supplementary Figure S1

Total ion chromatograms after P13ase digestion of HeLa cell extract at various pH values

Total ion chromatograms of digests obtained with P13ase at various pH values (pH 1.5 - 4.0) or with trypsin (pH 9.2). Chromatograms were analyzed with FreeStyle (Thermo Fisher Scientific).

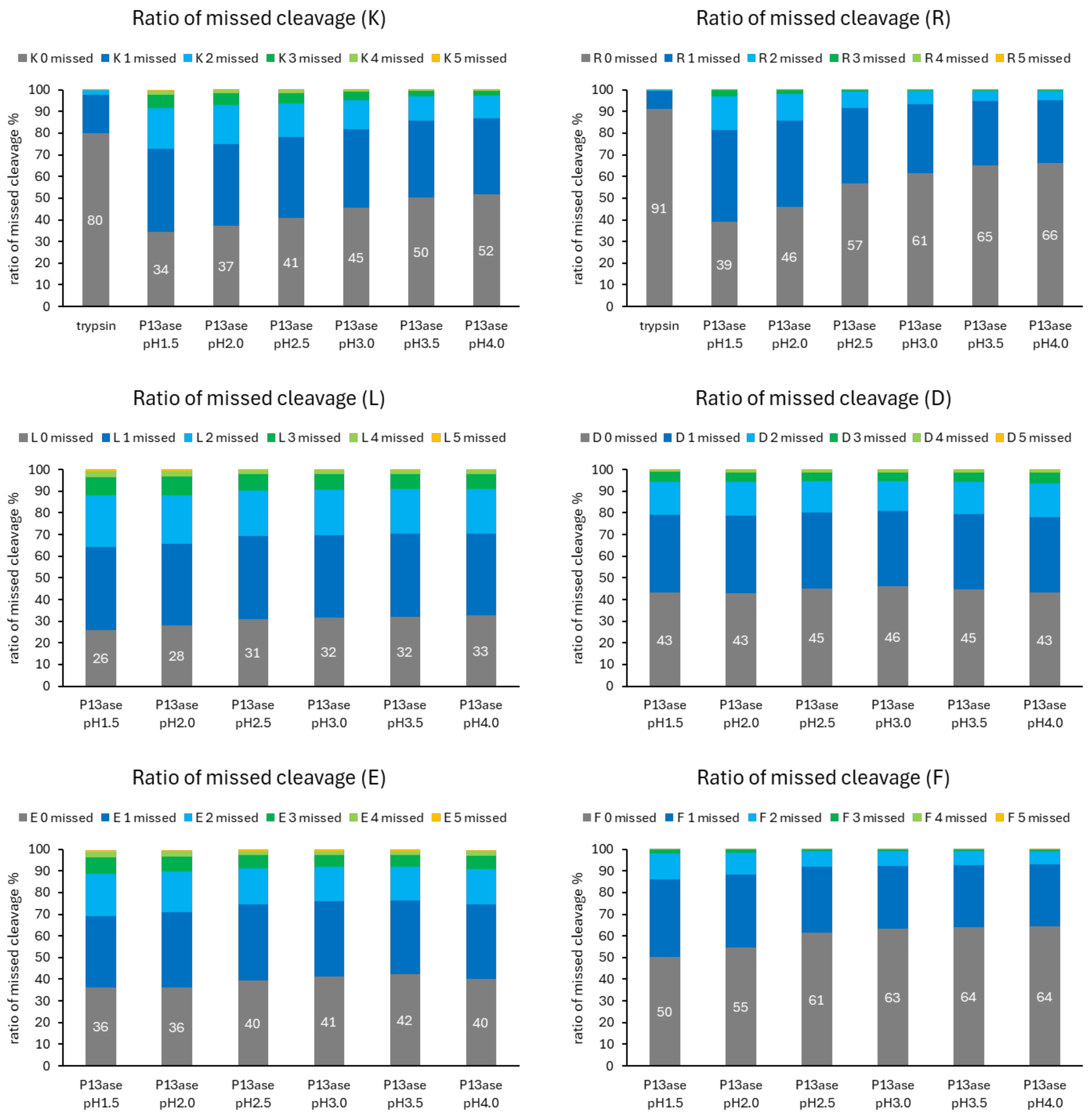

Supplementary Figure S2

Frequencies of missed cleavage by P13ase at various pH values

The missed cleavage frequencies of Lys, Arg, Leu, Phe, Asp and Glu residues by P13ase at various pH values (1.5 - 4.0). Frequencies were averaged from three technical replicates.

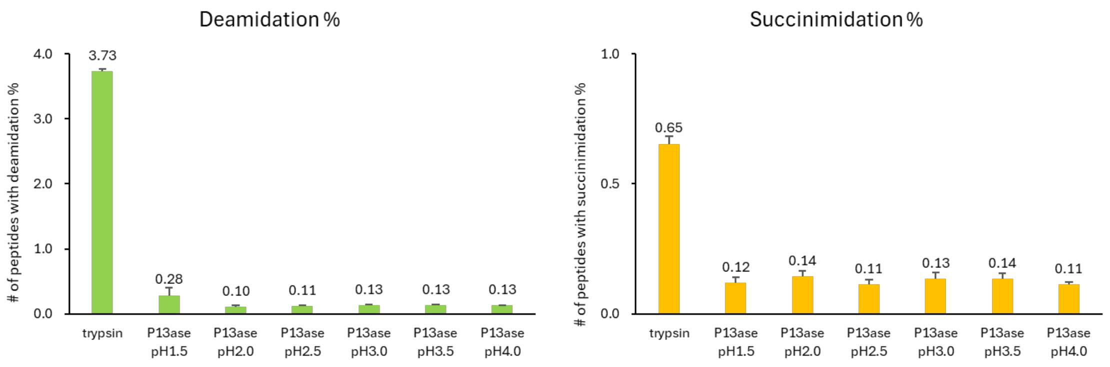

Supplementary Figure S3

Effect of digestion pH on artifactual deamidation and succinimidation

Comparison of the proportion of peptides containing deamidation or succinimidation after digestion with P13ase or trypsin at various pH values. The bar graphs represent the mean of three technical replicates and error bars represent the standard error.

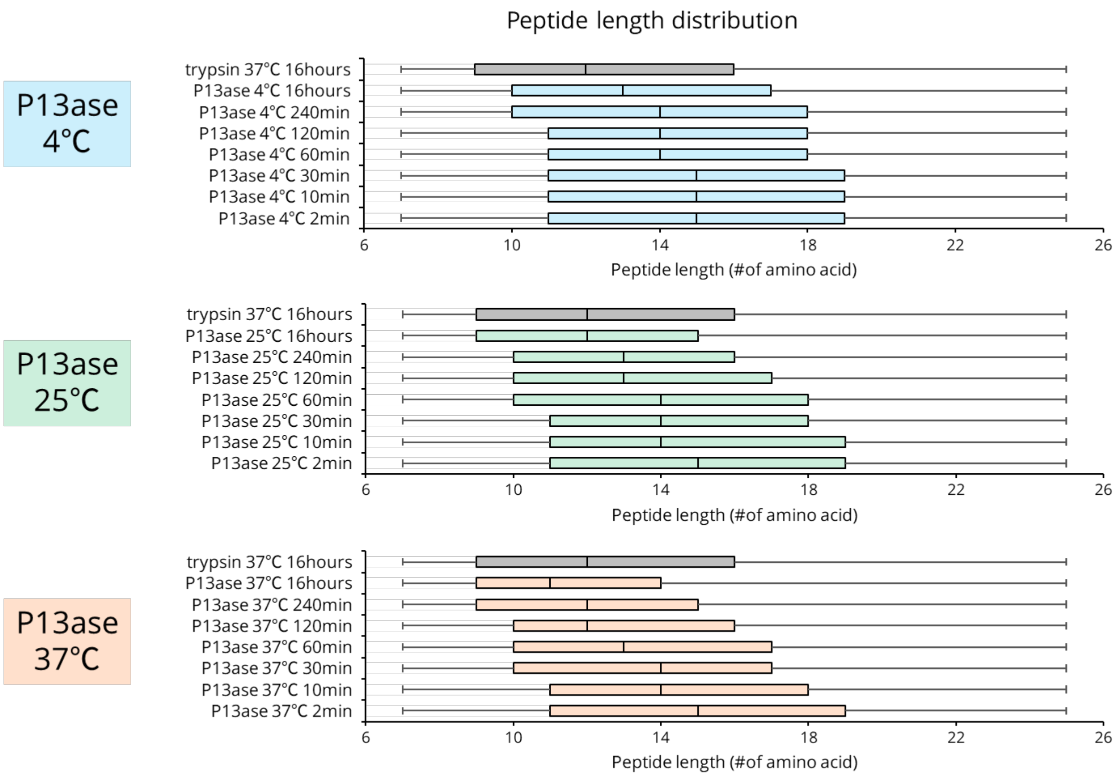

Supplementary Figure S4

Peptide lengths after P13ase digestion of HeLa cell extract for various times and at various temperatures

The length distribution of identified peptides after P13ase digestion for various times and at various temperatures.

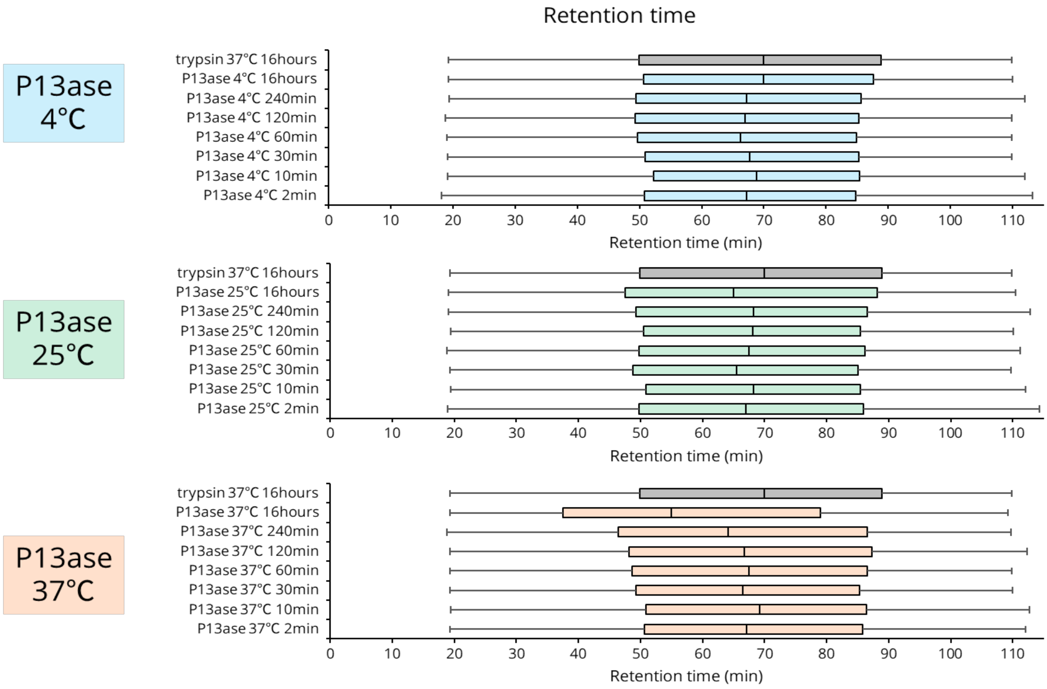

Supplementary Figure S5

Retention time distribution after P13ase digestion of HeLa cell extract for various times and at various temperatures

The distribution of retention times of identified peptides after P13ase digestion for various times and at various temperatures.

P13ase cleavage preference

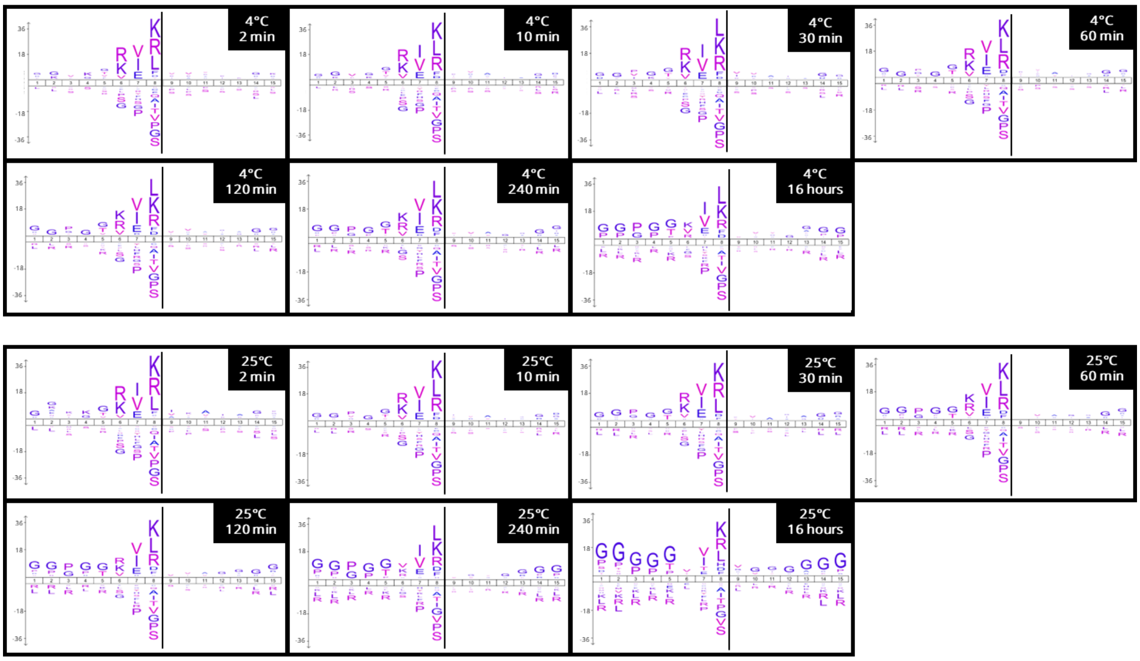

Supplementary Figure S6

Cleavage preferences of P13ase at various digestion times and temperatures

Cleavage preferences were visualized with iceLogo for various digestion times and temperatures. The y-axis is the percentage difference in frequency for an amino acid in the experimental set and the reference set.

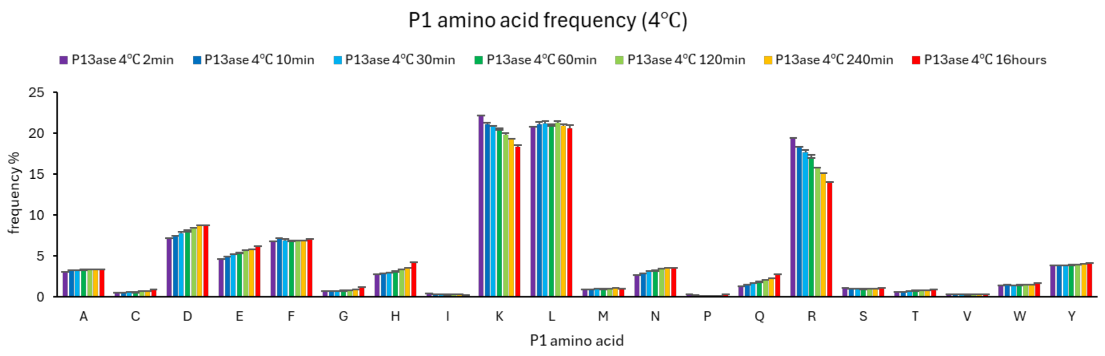

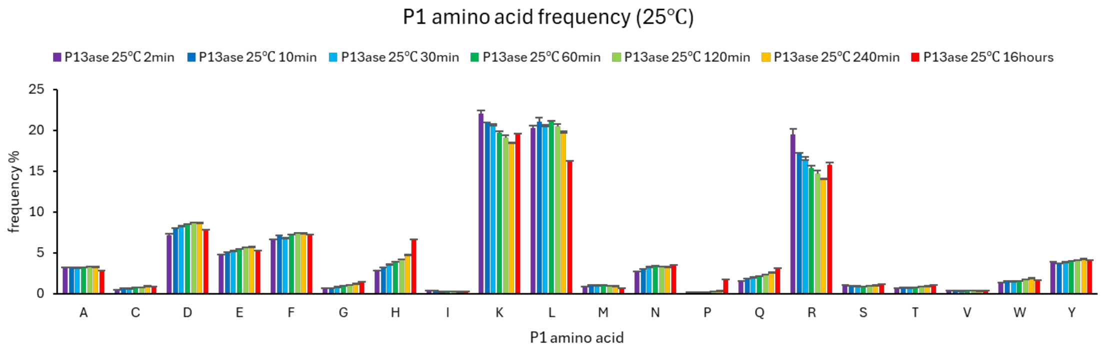

Supplementary Figure S7

Cleavage specificity of P13ase at various digestion temperatures and times

Frequencies of P1 amino acids at cleavage sites after digestion with P13ase at various temperatures for various times. The bar graphs represent the mean of three technical replicates and error bars represent the standard error.

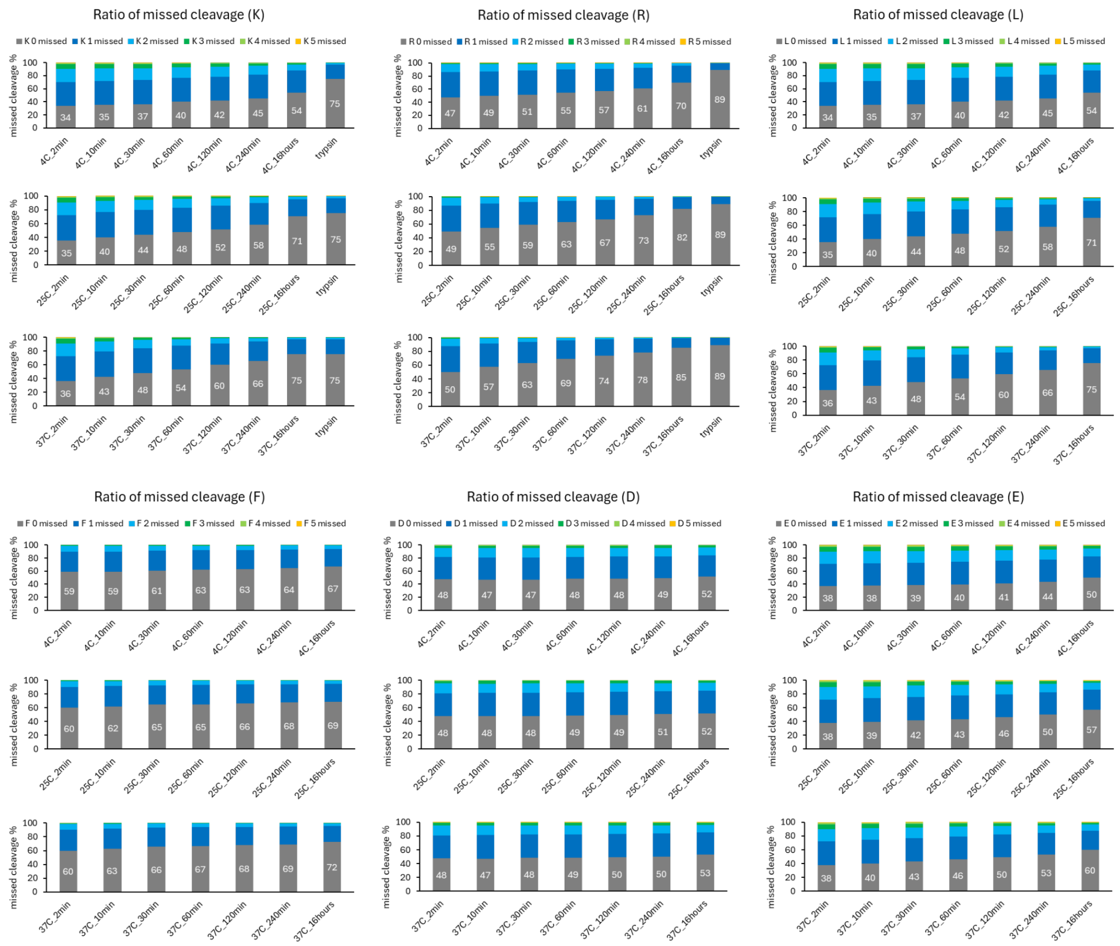

Supplementary Figure S8

Missed cleavage frequencies of P13ase at various digestion temperatures and times

The missed cleavage frequencies of Lys, Arg, Leu, Phe, Asp and Glu residues by P13ase at various digestion temperatures and times. Frequencies were averaged from three technical replicates.

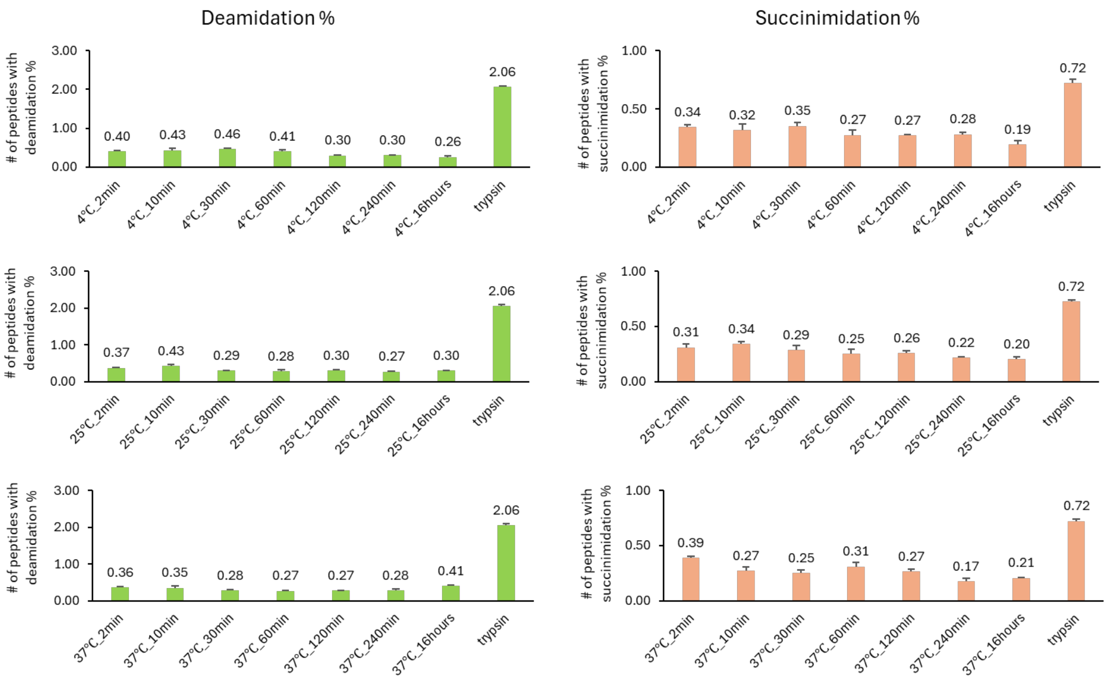

Supplementary Figure S9

Effect of digestion temperature and time on artifactual deamidation and succinimidation

Comparison of the proportion of peptides containing deamidation or succinimidation after digestion with P13ase or trypsin at various temperatures and for various times. The bar graphs represent the mean of three technical replicates and error bars represent the standard error.

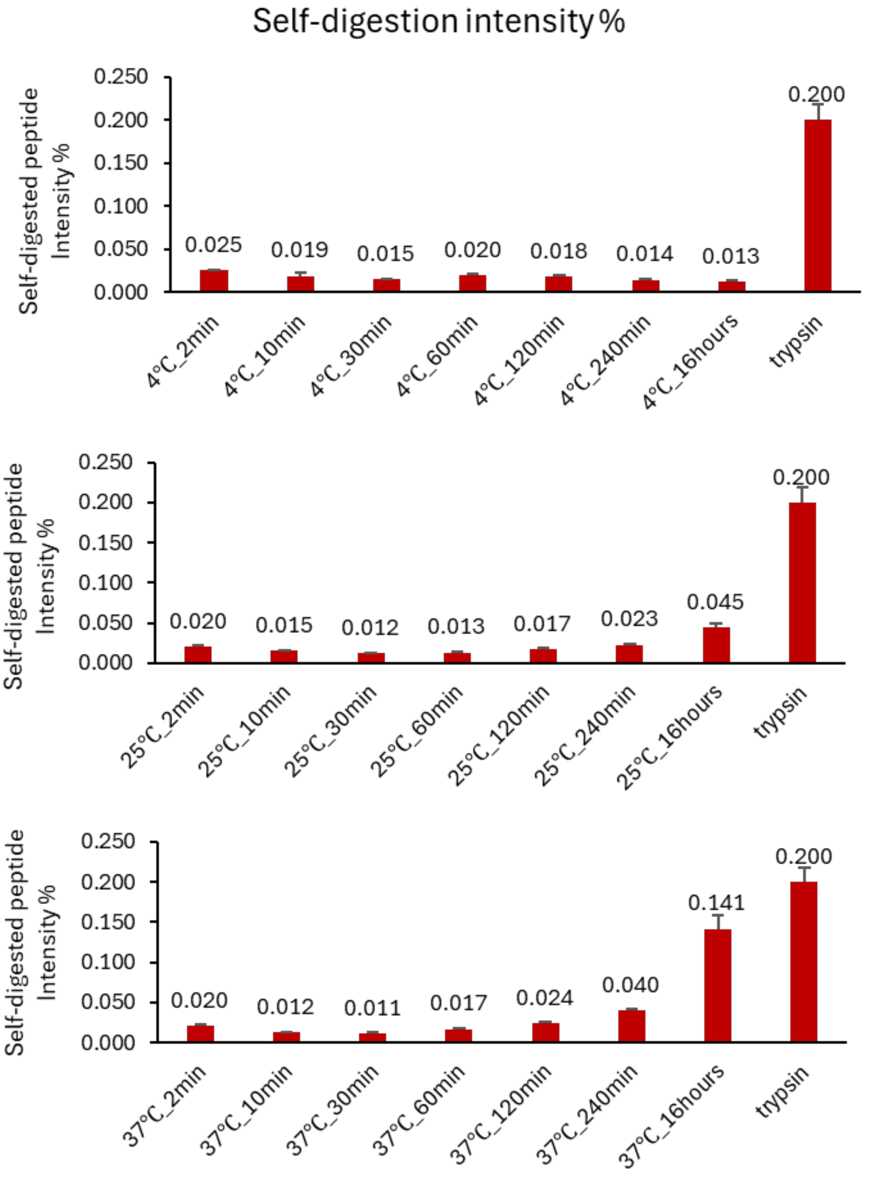

Supplementary Figure S10

Self-digested peptides generated by P13ase or trypsin

The ratios of summed intensity of self-digested identified peptides produced by P13ase or trypsin at various digestion temperatures and for various times. The bar graphs represent the mean of three technical replicates and error bars represent the standard error.

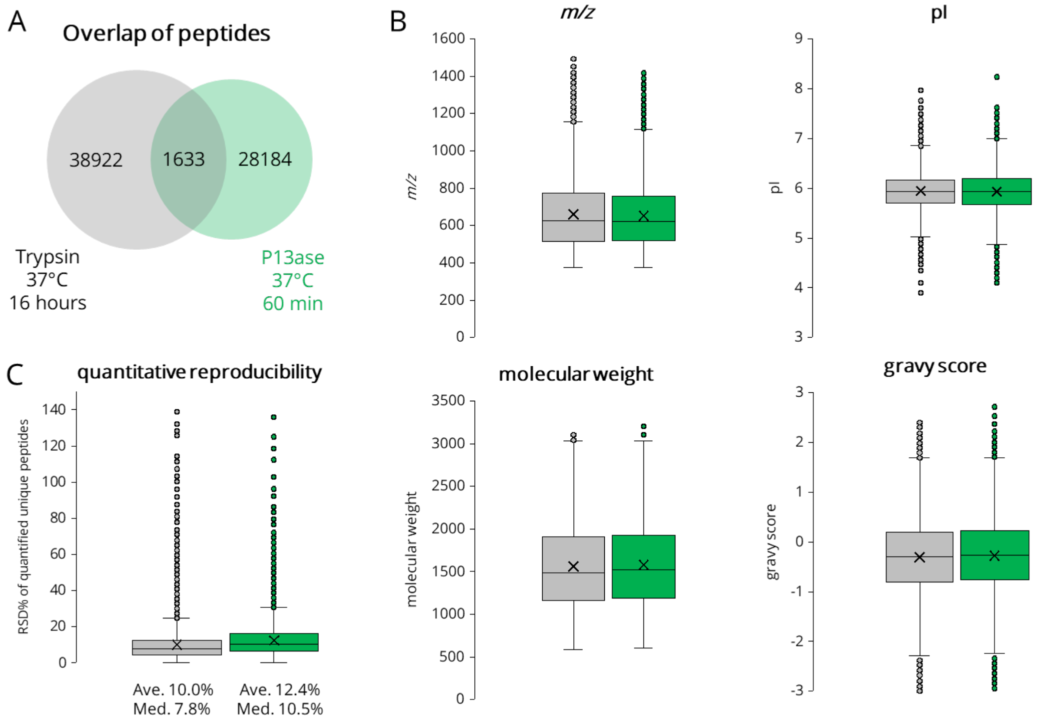

Supplementary Figure S11

Characteristics of peptides identified by P13ase digestion of HeLa cell extract

(A) Overlap of peptides identified by P13ase (37˚C, 60 min digestion) and trypsin (37˚C, 16 hours digestion). (B) Comparison of physicochemical properties (*m/z*, pI, molecular weight, gravy score) of peptides identified by P13ase and trypsin. (C) Quantitative reproducibility of identified peptides.

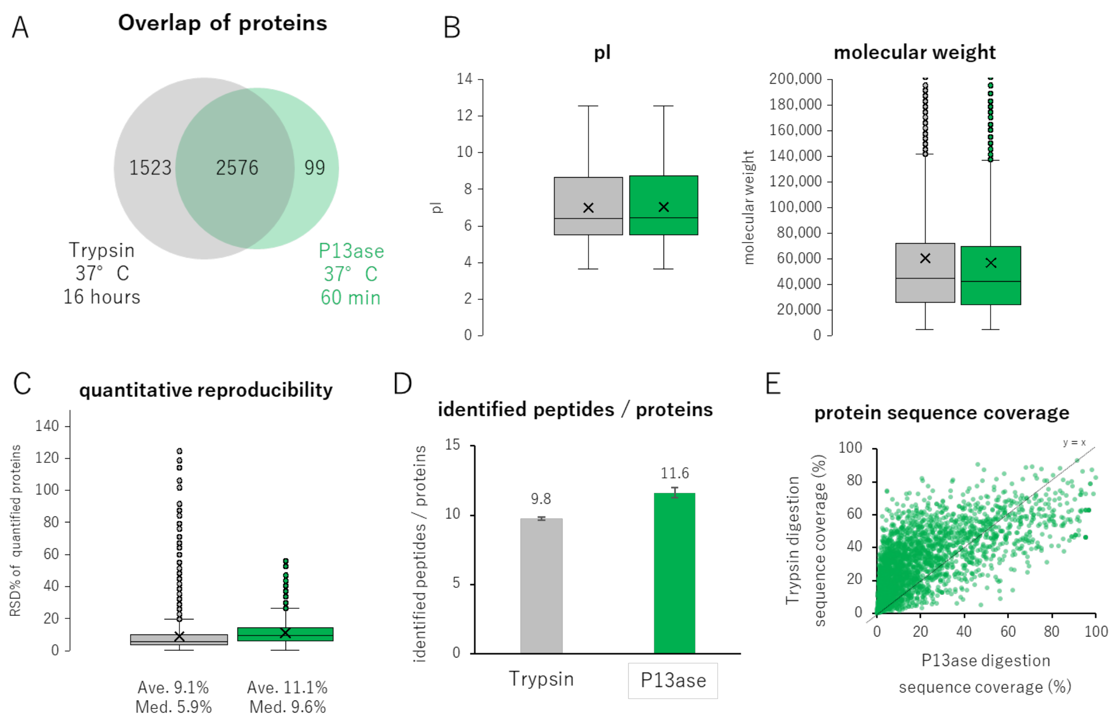

Supplementary Figure S12

Characteristics of proteins identified by P13ase digestion of HeLa cell extract

(A) Overlap of proteins identified by digestion with P13ase (37˚C, 60 min) and trypsin (37˚C, 16 hours). (B) Distributions of pI and molecular weights of proteins identified by P13ase and trypsin digestion. (C) Quantitative reproducibility of proteins identified by P13ase and trypsin digestion. (D) The number of peptides identified per protein by P13ase and trypsin digestion. The bar graphs represent the mean of three replicates, and error bars represent the standard error. (E) Scatterplot of sequence coverage of proteins commonly identified by P13ase and trypsin digestion. The sequence coverages were calculated with Protein Coverage Summarizer.

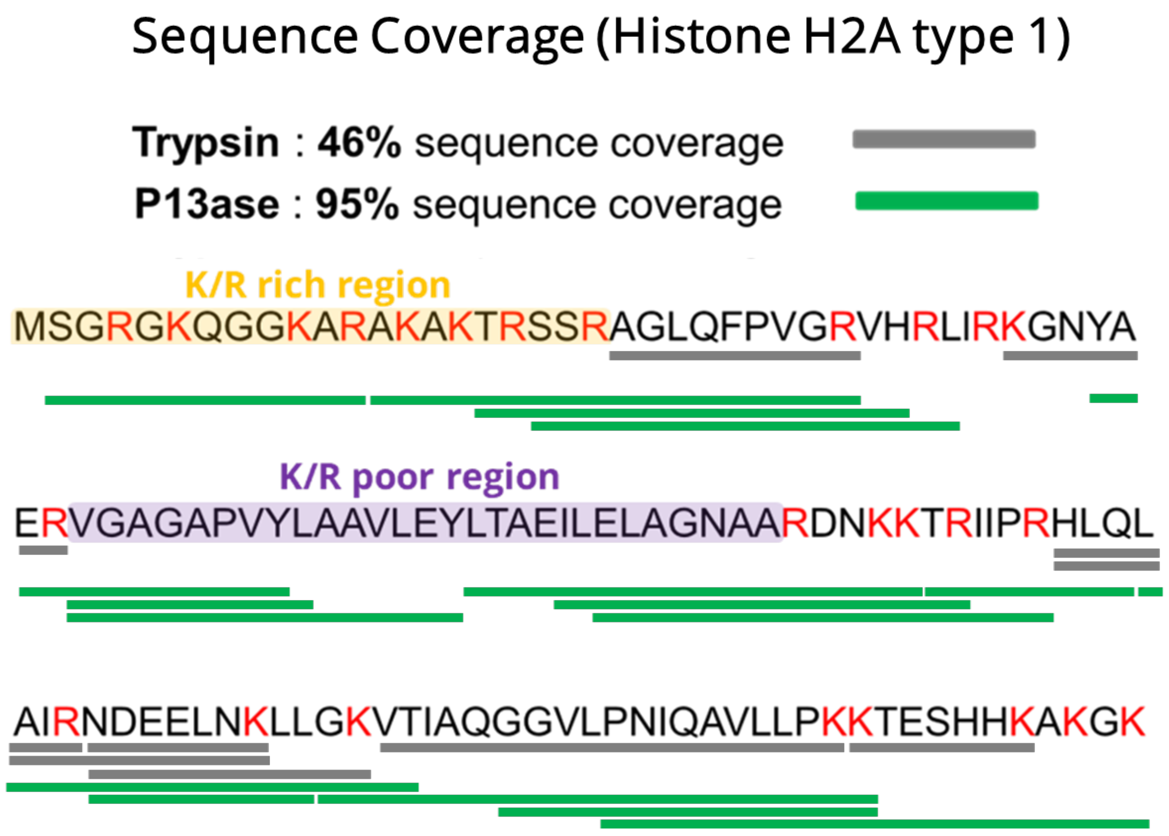

Supplementary Figure S13

Sequence coverage of histone H2A type 1 by P13ase or trypsin digestion

Sequence coverage of histone H2A type 1 by P13ase (37˚C, 60 min digestion) or trypsin (37˚C, 16 hours digestion). The sequence coverages were calculated with Protein Coverage Summarizer.

Supplementary Table S2
Motif analysis of the cleavage sites of P13ase (pH 3.5)

motif	Score	fold.increase	fg.matches	bg.matches
xxxxGxIL↓xxxxxxx	315.43	6.87	35	3213
xxxxGKxL↓xxxxxxx	314.76	6.57	35	3418
xxxxTxxL↓xxxxxxG	314.71	6.48	27	3089
xGxxxxIR↓xxxxxxx	314.40	8.37	22	1479
xxxxxVxR↓Gxxxxxx	314.28	7.49	28	2145
xxxGxxVL↓xxxxxxx	314.04	5.47	36	4364
GxxxxxIK↓xxxxxxx	313.90	9.48	30	1622
xxxxxRxL↓xxxxxxD	313.88	6.33	29	2996
xGxxxKxL↓xxxxxxx	313.77	6.87	28	2965
xxxxGxVL↓xxxxxxx	313.73	6.20	29	3820
xxxxxxIR↓xxxxxxx	307.65	3.38	120	23076
xxxxxxIK↓xxxxxxx	307.65	3.16	152	25249
xxxxxHxL↓xxxxxxx	307.65	2.76	90	27236
xxxxxxVR↓xxxxxxx	307.65	2.71	145	31376
xxxxxxVK↓xxxxxxx	307.65	2.54	152	34601
xxxxxxIL↓xxxxxxx	307.65	2.47	108	38607
xxxGxxxK↓xxxxxxx	307.65	2.42	108	28633
xxxxxKxL↓xxxxxxx	307.65	2.27	168	55690
xxxxxRxL↓xxxxxxx	307.65	2.11	196	62849

Motif analysis around the cleavage site (P8 ~ P7') was performed when rmotif-x was digested by P13ase at pH 3.5, 37°C, 10 min. Background was generated from FASTA used for database search. Those with a score of 300 or higher were extracted and listed. “ ↓ ” indicates cleavage sites.

Supplementary Table S3
Motif analysis of the cleavage sites of P13ase (37°C, 60 minutes)

motif	score	fold.increase	fg.matches	bg.matches
xxxxGKxL↓xxxxxxx	318.1	7.25	50	3621
xxxGxxVL↓xxxxxxx	316.6	5.77	47	4374
xxxxGxIL↓xxxxxxx	316.1	6.91	38	3010
xxxPxxVK↓xxxxxxx	315.8	6.87	36	1993
xGxxxxIR↓xxxxxxx	315.3	8.07	26	1479
GxxxxxIR↓xxxxxxx	315.0	7.69	26	1579
xxPxxxxK↓xxxxVxx	314.9	8.11	25	1532
xxxxxVxR↓Gxxxxxx	314.1	7.78	25	1817
xGxxxxIK↓xxxxxxx	314.1	7.14	25	1382
xxGxxxIK↓xxxxxxx	313.9	8.45	29	1331
xGxxxxxL↓xxVxxxx	313.7	5.01	36	4001
xxxxxxIK↓xxxxxxx	307.7	2.66	113	21570
xxxGxxxK↓xxxxxxx	307.7	2.31	184	31885
xxxxGxxK↓xxxxxxx	307.7	2.29	136	27506
xxxxxxVK↓xxxxxxx	307.7	2.16	153	30637
xxxxxxVR↓xxxxxxx	307.7	2.58	147	29396
xGxxxxxR↓xxxxxxx	307.7	2.33	118	31327
xxxGxxxR↓xxxxxxx	307.7	2.09	173	39431
GxxxxxxL↓xxxxxxx	307.7	1.99	227	64575
xxGxxxxL↓xxxxxxx	307.7	1.97	201	61619
xxPxxxxL↓xxxxxxx	307.7	1.97	177	58236
xxxxxKxL↓xxxxxxx	307.7	2.15	157	50547
xxxxxRxL↓xxxxxxx	307.7	2.08	151	54233
xxxxGxxL↓xxxxxxx	307.7	2.34	124	42924
xxxxxxIL↓xxxxxxx	307.7	2.96	113	33596
xxxxTxxL↓xxxxxxx	307.7	2.60	97	36364

Motif analysis around the cleavage site (P8 ~ P7') was performed when rmotif-x was digested by P13ase at pH 3.5, 37°C, 60 min. Background was generated from FASTA used for database search. Those with a score of 300 or higher were extracted and listed. " ↓ " indicates cleavage sites.

Supplementary Table S4
Motif analysis of the cleavage site of P13ase (37°C, 16 hours)

motif	score	fold.increase	fg.matches	bg.matches
GGxxxxxR↓xxxxxxx	314.2	14.65	20	3333
GxxxxxxL↓xxxxxxx	307.7	4.78	65	65520
xxxxGxxL↓xxxxxxx	307.7	4.77	45	60461
xxGxxxxR↓xxxxxxx	307.7	4.02	59	38761
xxxxxxxK↓xxxxxxG	307.7	3.84	62	35502

Motif analysis around the cleavage sites (P8 ~ P7') was performed when rmotif-x was digested by P13ase at pH 3.5, 37°C, 16 hours. Background was generated from FASTA used for database search. Those with a score of 300 or higher were extracted and listed. " ↓ " indicates cleavage sites.
